# P02.36. Meditation or exercise for preventing acute respiratory infection: a randomized controlled trial

**DOI:** 10.1186/1472-6882-12-S1-P92

**Published:** 2012-06-12

**Authors:** B Barrett, D Rakel, M Hayney, D Muller, A Zgierska, C Obasi, T Ewers, R West, R Brown, Z Zhang, M Gassman, S Barlow, C Coe

**Affiliations:** 1 University of Wisconsin, Madison, USA

## Purpose

This study was designed to evaluate potential preventive effects of meditation or exercise on incidence, duration, and severity of acute respiratory infection (ARI) illness.

## Methods

Community-recruited adults aged ≥ 50 years were randomized to one of three conditions: 8-week training in mindfulness meditation; matched 8-week training in moderate intensity sustained exercise; or wait-list observational control. The primary outcome was area-under-the-curve global illness severity over one cold and flu season, using the Wisconsin Upper Respiratory Symptom Survey (WURSS-24) to assess severity. Significance was set at p=0.025. Health care visits and days-of-missed-work were counted. Nasal wash collected during ARI illness was assayed for neutrophils, interleukin-8, and viral nucleic acid.

## Results

Of 154 randomized, 149 completed the trial (82% female, 94% white, mean age 59.3 ± SD 6.6 years). There were 27 ARI episodes and 257 days of ARI illness in the meditation group (n=51), 26 episodes and 241 illness days for exercise (n=47), and 40 episodes and 453 days for control (n=51). Mean global severity was 144 for meditation, 248 for exercise, and 358 for control. Compared to control, global severity was significantly lower for meditation (p=0.0042). Both global severity and total days of illness (duration) trended towards being lower for exercise (p=0.16 and p=0.032, respectively), as did duration for the meditation group (p=0.034). Adjusting for covariates using zero-inflated multivariate regression models gave similar results. There were 67 ARI-related days-of-missed-work in the control group, 32 in the exercise group (p=0.041), and 16 for meditation (p<0.001). Healthcare visits did not differ significantly. Viruses were identified in 54% of samples from meditation, 42% from exercise, and 54% from control. Neutrophil count and interleukin-8 levels were similar among intervention groups.

## Conclusion

Training in meditation or exercise may be effective in reducing ARI illness burden.

